# Preparation and Electrochemical Performance of Na_2−x_Li_x_FePO_4_F/C Composite Cathode Materials with Different Lithium/Sodium Ratios

**DOI:** 10.3390/mi15010015

**Published:** 2023-12-21

**Authors:** Lei Wang, Hualing Tian, Xiang Yao, Yanjun Cai, Ziwei Gao, Zhi Su

**Affiliations:** 1 College of Chemistry and Chemical Engineering, Xinjiang Normal University, Urumqi 830054, China; wanglei@xjnu.edu.cn (L.W.); tianhuali1979@sina.cn (H.T.); yxiangxjnu@163.com (X.Y.); caiyanjun1976@sina.cn (Y.C.); 2 Xinjiang Key Laboratory of Energy Storage and Photoelectrocatalytic Materials, Urumqi 830054, China; 3 Shanxi Key Laboratory of Chemical Reaction Engineering, College of Chemistry & Chemical Engineering, Yan’an University, Yan’an 716000, China; 4 Key Laboratory of Applied Surface and Colloid Chemistry, Ministry of Education, Xi’an Key Laboratory of Organometallic Material Chemistry, School of Chemistry & Chemical Engineering, Shanxi Normal University, Xi’an 710119, China; 5 College of Energy and Chemical Engineering, Xinjiang Institute of Technology, Akesu 843100, China

**Keywords:** sodium-ion battery, cathode material, Na_2−x_Li_x_FePO_4_F, electrochemical performance

## Abstract

With their advantages of abundant raw material reserves, safety, and low toxicity and cost, sodium-ion batteries (SIBs) have gained increasing attention in recent years. Thanks to a high theoretical specific capacity (124 mAh g^−1^), a high operating voltage (about 3.2 V), and a very stable three-dimensional layered structure, sodium ferric fluorophosphate (Na_2_FePO_4_F, NFPF) has emerged as a strong candidate to be used as a cathode material for SIBs. However, applications are currently limited due to the low electronic conductivity and slow ion diffusion rate of NFPF, which result in a low actual specific capacity and a high rate performance. In this study, the authors used a high-temperature solid-phase technique to produce Na_2−x_Li_x_FePO_4_F/C (0 ≤ x ≤ 2) and evaluated the impact on electrode performance of materials with different Na^+^ and Li^+^ contents (values of x). Transmission electron microscopy (TEM) and X-ray diffraction (XRD) were also used to analyze the material’s crystal structure and nanostructure. The results show that the material had the best room-temperature performance when x = 0.5. At a charge–discharge rate of 0.1 C, the first discharge-specific capacity of the resulting Na_1.5_Li_0.5_FePO_4_F/C cathode material was 122.9 mAh g^−1^ (the theoretical capacity was 124 mAh g^−1^), and after 100 cycles, it remained at 118 mAh g^−1^, representing a capacity retention rate of 96.2% and a Coulomb efficiency of 98%. The findings of this study demonstrate that combining lithium and sodium ions improves the electrochemical performance of electrode materials.

## 1. Introduction

In the 1970s, sodium-ion materials were considered alongside lithium-ion materials for use in batteries. As both are alkali metals, they share a lot of physical and chemical properties. However, the greater radius of sodium ions (0.102 nm) compared to lithium ions (0.076 nm) contributes to difficulties related to the reversible deintercalation of sodium ions in electrode materials, and even if deintercalation does occur, it has slow diffusion kinetics [1]. Ultimately, this research did not lead to the wide-scale exploitation of sodium-ion batteries (SIBs) due to the irreversible phase transition of the electrode material during the charge–discharge process. In the decades since moving away from fossil fuels and toward sustainable manufacturing and energy use, the demand for lithium-ion batteries (LIBs) has increased exponentially. However, the scarcity and uneven distribution of lithium resources have created a critical need to develop next-generation secondary batteries that can wholly or partially replace lithium-ion batteries. With their advantages, such as the abundance of sodium resources, low cost, and good safety [2], interest in sodium-ion batteries has been reignited. As a result, SIBs have been identified as the secondary batteries that are most likely to replace lead acid batteries or partially replace LIBs in large-scale energy storage and low-speed electric vehicles.

SIBs rely on the same “rocking-chair” mechanism as LIBs to convert energy, reversibly extracting/intercalating Na^+^ between the positive and negative electrodes during charging and discharging. As a result, the intrinsic chemical characteristics of the electrode materials have a significant impact on the electrochemical performance of SIBs. The quality of the cathode material also determines the output voltage of the battery, making it the primary factor that directly influences the electrochemical performance of SIBs. Materials currently being investigated as possible cathode materials for SIBs include transition metal oxides, polyanions, derivatives of Prussian blue, and other organic compounds, all of which have specific capacities of 120 to 200 mAh/g [3]. Compared to other cathode materials, polyanionic compounds have become widely studied electrode materials due to their diverse structures, high safety, and excellent electrochemical performance. At present, the main polyanion compounds that have been studied include phosphate, pyrophosphate, fluoride phosphate, and sulfate. Among polyanionic compounds, NASICON-type compounds are widely considered the most promising cathode materials for sodium-ion batteries because of their open structure and unblocked sodium ion diffusion channels. The layered iron-based fluorophosphate of Na_2_FePO_4_F is particularly attractive by virtue of its facile 2D Na^+^ pathways formed by interconnected PO_4_ tetrahedra and FeO_4_F_2_ octahedra, its great structural stability with small volume variations (only 3.7%) during sodiation/desodiation, the huge availability of iron ore reserves, its satisfactory theoretical capacity (124 mA·h·g^−1^), and the decent operating voltage (≈3.0 V vs. Na^+^/Na) induced by the polyanion effect [4,5]. Most current Na_2_FePO_4_F research focuses on the material’s structure and charge–discharge mechanism. For instance, Lui et al. investigated the migration mechanism of sodium ions during the charging process using in situ XRD and NMR techniques in a study that gave designers of modified polyanionic cathode materials a preliminary theoretical foundation to build on [6]. Through theoretical calculations, Ellis et al. predicted the migration path of sodium ions in Na_2_FePO_4_F and the change in volume after charging [7].

However, like other cathode materials, Na_2_FePO_4_F faces three main problems that severely restrict its practical use as a cathode material for SIBs: poor cycle stability, limited sodium storage utilization, and low electrical conductivity. Currently, ion doping and carbon coating are the two methods available to tackle these issues [8]. Doping can significantly increase the sodium ion diffusion coefficient of a single particle, while carbon coating can lower the charge resistance at the interface between two neighboring particles, as well as between the electrolyte and the particles themselves [9]. Most research to date has focused on improving the electrochemical properties of the materials via polyanion coating, doping, and the use of various carbon sources. There has been little research and exploration on the introduction of lithium ions into materials.

In this study, electrode materials with the general formula Na_2−x_Li_x_FePO_4_F/C (0 ≤ x ≤ 2) were prepared, and the influence of the addition of lithium ions on the cathode material was explored by controlling the different proportions of lithium ions. Ultimately, it was found that the Na_1.5_Li_0.5_FePO_4_F/C material had the best electrochemical performance. A high-temperature solid-state method was used to create the Na_2−x_Li_x_FePO_4_F/C (0 ≤ x ≤ 2) cathode material. X-ray diffraction (XRD) and transmission electron microscopy (TEM) were used to investigate the structural composition, and cyclic charge–discharge tests were utilized to investigate the electrochemical performance. The crystal structure of Na_2−x_Li_x_FePO_4_F/C (0 ≤ x ≤ 2) was found to transition from Pbcn to Pnma with the addition of Li^+^ ions in different stoichiometric ratios. The electrochemical capacity gradually increased with increasing lithium contents and reached the optimum value of 122.9 mAh g^−1^ for Na_1.5_Li_0.5_FePO_4_F/C (x = 0.5). As the Li^+^ content was increased further, the electrochemical capacity gradually decreased again due to the excessive exchange of lithium and sodium ions during the reaction process, resulting in excessive Na^+^/Li^+^ ion mixing and greater internal resistance, which affected the electrochemical capacity of the material [10].

## 2. Result and Discussion

**Figure 1:**
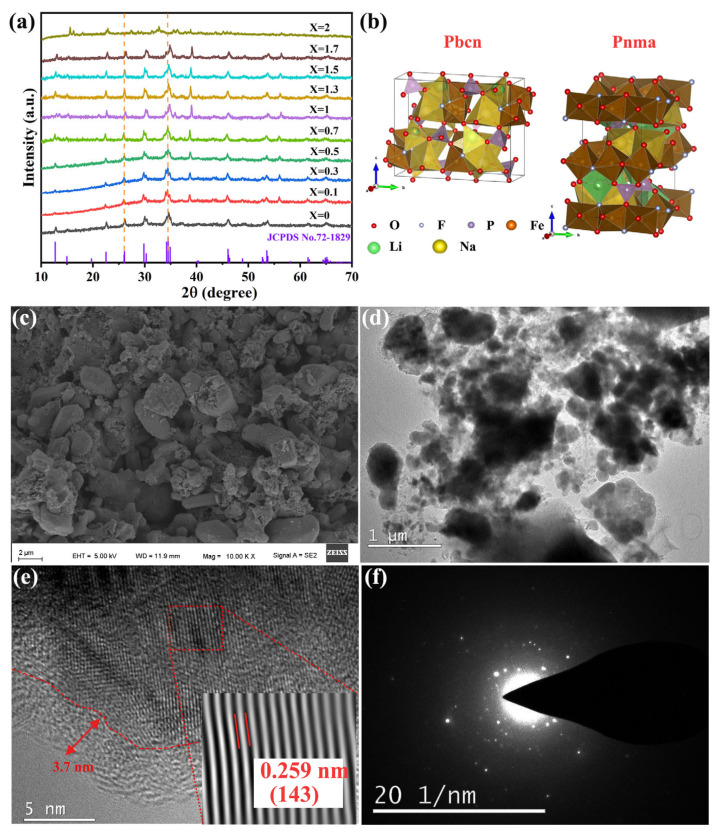
(**a**) XRD patterns of the Na_2−x_Li_x_FePO_4_F/C samples, (**b**) different structures of Na_1.5_Li_0.5_FePO_4_F/C materials (Pbcn and Pnma), (**c**) SEM images, (**d**) TEM image, (**e**) HR-TEM image, and (**f**) SAED images of the Na_1.5_Li_0.5_FePO_4_F/C sample.

X-ray diffraction (XRD) was used to examine the effects of different degrees of lithium doping on the crystal structures of the composites. The XRD patterns of the as-prepared Na_2−x_Li_x_FePO_4_F/C (0 ≤ x ≤ 2) samples are shown in Figure 1a. The 2*θ* range was 10–70, and the scanning speed was 7° min^−1^. With a gradual increase in X (increase in the lithium content), the diffraction peaks moved to larger angles, indicating that the material’s interplanar spacing gradually decreased. This was because the lithium-ion radius (0.076 nm) is smaller than the sodium-ion radius (0.102 nm) and, as a result, the material’s unit cell volume and the interplanar spacing got smaller as lithium ions replaced sodium ions. The main diffraction peaks of the Na_2−x_Li_x_FePO_4_F/C materials roughly corresponded to the standard card (PDF#72-1829), indicating that Li^+^ had successfully penetrated the material’s crystal lattice. The unit cell data of the as-prepared materials (Table S2) clearly show that as the X value increased, the unit cell volume decreased. At the same time, the unit cell space group changed from Pbcn (Na_2_FePO_4_F/C) to Pnma (Na_1.5_Li_0.5_FePO_4_F/C), indicating that the addition of lithium ions had a significant influence on the crystal structure of the material and that the unit cell volume shrank (Figure 1b) [11].

Figure 1c–f show SEM and TEM images of Na_1.5_Li_0.5_FePO_4_F/C. The SEM image (Figure 1c) shows a relatively uniform particle distribution with occasional particle agglomeration. The particle surface is relatively loose and rough, accompanied by pore structures, with an average particle distribution of about 1 μm. This morphology indicates that the COx gas generated during the preparation process promotes the formation of pore structures. This is supported by a study of the three-dimensional interconnected porous structure of sodium-ion batteries by Didwal et al. [12]. TEM images of the morphology and microstructure of the Na_1.5_Li_0.5_FePO_4_F/C material show that the material consists of many particles (Figure 1d). The material is wrapped in a uniform carbon layer with a thickness of about 4.0 nm formed by high-temperature calcination and carbonization of glucose, which can effectively prevent agglomeration and buffer the stress generated by the grain growth of the material [13], effectively improving the electronic conductivity and electrochemical performance of the material. The lattice fringes of the material are also clearly visible, indicating a high degree of crystallinity in the sample. The corresponding Na_1.5_Li_0.5_FePO_4_F/C (143) crystal plane distance is consistent with the lattice distance of 0.259 nm. Granular materials with nanometer characteristics have good electronic conductivity and high-rate charge–discharge performances (Figure 1e). Figure 1f is the selected electron diffraction pattern of the material, and it can be seen that the material has polycrystalline characteristics.

**Figure 2:**
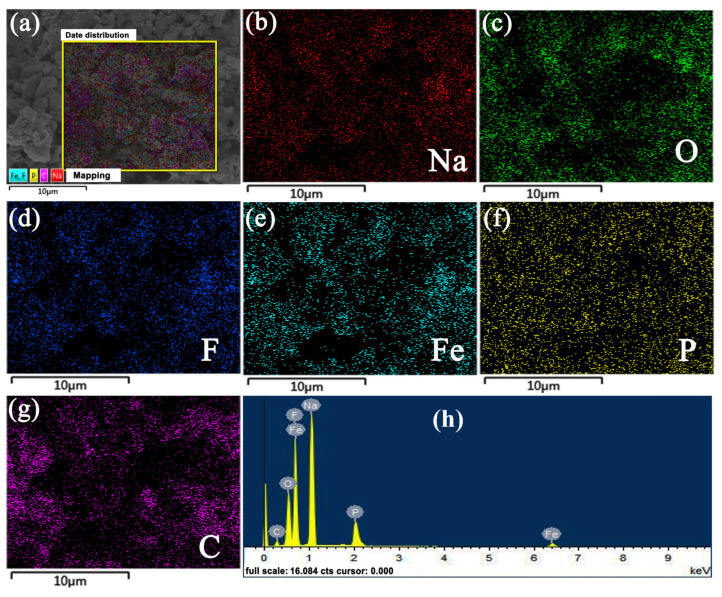
(**a**) Elemental mapping of Na_1.5_Li_0.5_FePO_4_F/C. (**b**) Na, (**c**) O, (**d**) F, (**e**) Fe, (**f**) P, (**g**) C, (**h**) EDS spectrum.

The elemental mapping of the Na_1.5_Li_0.5_FePO_4_F/C material is shown in Figure 2. The elements C, F, O, P, Fe, and Na are evenly distributed in the sample. Furthermore, the ferric sodium fluophosphate material is coated with a thin layer of carbon, effectively improving the material’s conductivity.

**Figure 3:**
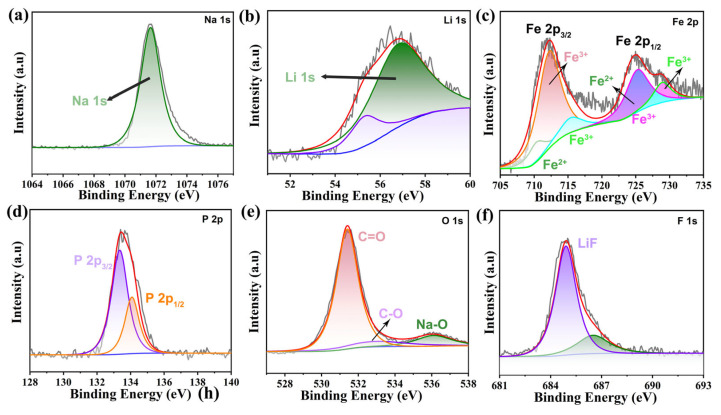
The high-resolution XPS spectra of (**a**) Na 1s, (**b**) Li 1s, (**c**) Fe 2p, (**d**) F 1s, (**e**) P 2p, and (**f**) O 1s of Na_1.5_Li_0.5_FePO_4_F/C.

The Na_1.5_Li_0.5_FePO_4_F/C material was characterized via X-ray photoelectron spectroscopy (XPS), and it was found, based on the total spectrum, that the samples mainly contained Na, Li, Fe, P, O, and F elements (Figure 3). It is well known that the characteristic peak of the Na 1s high-resolution spectrum at 1071.6 eV proves the formation of the metallic Na phase at the surface (Figure 3a). The high-resolution spectrogram of the Li 1s (Figure 3b) shows two peaks at 54.8 and 56 eV, indicating that the material contains Li^+^. Figure 3c shows that the Fe 2p spectrum can be decomposed into two groups of Fe species with Fe^2+^ and Fe^3+^ characteristics and two weak satellite peaks (Sat.). Fitting peaks with combined energies of 710.3 eV and 724.1 eV are labeled Fe^2+^, while peaks at 712.2 eV, 715.1 eV, 725.2 eV, and 728.9 eV are attributed to Fe^3+^. Figure 3d shows the P 2p spectrum of the material, forming two peaks at 133.4 eV and 134.4 eV. The peak at 134.4 eV is in the form of metal–phosphorus bonds, and the peak at 133.4 eV can be attributed to the oxidized phosphorus and the phosphate formed on the surface. Figure 3e shows the peaks observed in the O 1s spectrum in the matrix of metallic compounds as lattice oxygen (529.8 eV) and oxygen vacancy (531.5 eV). As shown in Figure 3f, the fitted peak at 687.7 eV in the XPS profile of the F element is attributed to the chemical interaction of fluorine with the crystal, while the fitted peak at 685.0 eV is in the metal fluoride region, so it is reasonable to attribute it to metal fluoride, indicating the presence of F^-^ in this sample. The above results show that a new type of Na_1.5_Li_0.5_FePO_4_F/C nanomaterial was successfully prepared, and its abundant valence states will play an important role in electrochemical applications.

**Figure 4:**
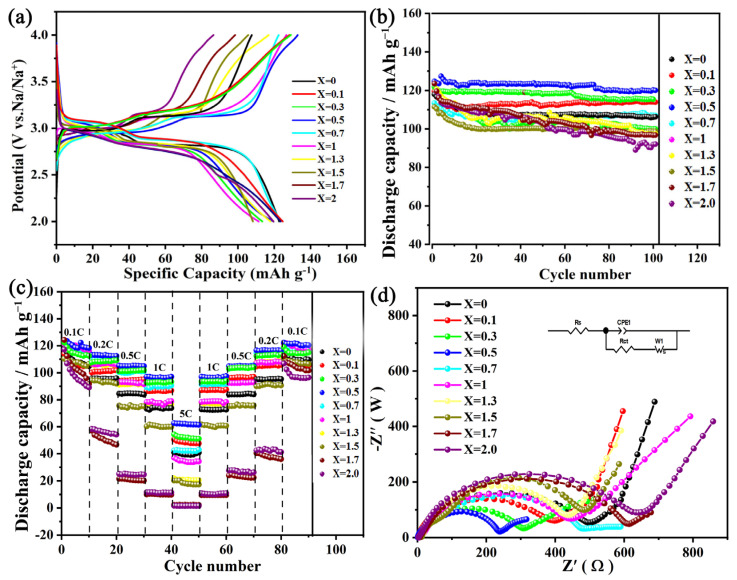
(**a**) The first-cycle charge–discharge curves of the Na_2−x_Li_x_FePO_4_F/C (0 ≤ x ≤ 2) materials, (**b**) cycle performance diagram of the Na_2−x_Li_x_FePO_4_F/C (0 ≤ x ≤ 2) materials, (**c**) magnification diagram of the Na_2−x_Li_x_FePO_4_F/C (0 ≤ x ≤ 2) materials, and (**d**) impedance diagram of the Na_2−x_Li_x_FePO_4_F/C (0 ≤ x ≤ 2) materials.

Figure 4a shows the first-cycle charge–discharge curve of Na_2−x_Li_x_FePO_4_F/C (0 ≤ x ≤ 2). There are two charge–discharge plateaus at 3.04 V and 2.86 V, indicating that the electrochemical process includes two deintercalations. In the first week after the Na_2−x_Li_x_FePO_4_F/C (0 ≤ x ≤ 2) samples were prepared, the charge–discharge capacity gradually increased with the continuous increase in the lithium content, from 117 mAh g^−1^ for unmixed Li^+^ to the maximum value of 122.9 mAh g^−1^ (Na_1.5_Li_0.5_FePO_4_F/C), and then gradually decreased with the further increase in Li^+^ (Table S3). The initial Coulombic efficiency of the Na_1.5_Li_0.5_FePO_4_F/C materials reached 92.7%. The electrochemical properties of the materials showed corresponding characteristics. This performance may be due to two factors. The first is that as the Li^+^ content increases, the unit cell of the cathode material changes from Pbcn to Pnma [14]. The results of the experiments also show that all samples are electrochemically active, indicating that Na^+^/Li^+^ ion exchange occurs gradually and that the unit cell volume changes accordingly [15]. This explains the gradual increase in the electrochemical capacity after the Li^+^ is first added. The second factor governing this performance is that as the Li^+^ content increases, the material’s electrochemical capacity gradually decreases, which may be related to the large ion mixing after the continuous Na^+^/Li^+^ ion exchange [16]. The internal resistance of the material also increases excessively. This is related to the impedance, which is discussed below. The results show that the optimal Na_2−x_Li_x_FePO_4_F/C (0 ≤ x ≤ 2) ratio for cathode materials for use in hybrid sodium-ion batteries is Na_1.5_Li_0.5_FePO_4_F/C.

The cycling performance of Na_2−x_Li_x_FePO_4_F/C (0 ≤ x ≤ 2) at 0.1 C with a voltage range of 2 V to 4 V and differing Li^+^ contents is shown in Figure 4b. For x = 0, 0.1, 0.3, 0.5, 0.7, 1, 1.3, 1.5, 1.7, and 2, the material’s capacity retention rates after 100 cycles were measured to be 95.2%, 80.6%, 78.3%, 96.2%, 74.2%, 78%, 94.7%, 89.9%, 94.2%, and 88.4%, respectively, showing that the capacity retention rate of the electrode material was greatest when x = 0.5. When the lithium-ion content is too low, it has little effect on the material, while properly increasing the lithium-ion content can significantly improve cycling performance and stability. The lattice parameters of Na_1.5_Li_0.5_FePO_4_F are a = 10.9348(3) Å, b = 6.3564(6) Å, 11.5689(4) Å, and V = 812.31(2) Å^3^. A comparative analysis of the lattice data of Na_1.5_Li_0.5_FePO_4_F and NaLiFePO_4_F (Table S2) shows that the lattice volume of NaLiFePO_4_F is only V = 801.7(5) Å^3^, a reduction that is related to the substitution of sodium ions (*R* = 0.102 nm) with lithium ions with smaller ionic radii (*R* = 0.076 nm) [11]. However, an excessive lithium-ion content reduces the electrochemical performance and stability of the material due to ion mixing. Figure 4c shows a magnification diagram of the Na_2−x_Li_x_FePO_4_F/C (0 ≤ x ≤ 2) materials. At all current densities ranging from 0.1 C to 5 C, the rate performance of the hybrid cathode material with x = 0.5 (Na_2−x_Li_x_FePO_4_F/C) is the best, with a reversible discharge capacity of 62.6 mAh g^−1^ at 5 C and an initial capacity retention rate of 97.8% when returning to 0.1 C after 80 cycles at different rates.

Figure S1a–d show cyclic voltammograms for four mixed materials: Na_2_FePO_4_F/C Na_1.5_Li_0.5_FePO_4_F/C, NaLiFePO_4_F/C, and NaLi_2_FePO_4_F/C. The CV curves of all four materials have good overlap, demonstrating the excellent reversibility of the materials. The CV peaks of the three mixed materials are sharper than those of the unmixed material, demonstrating an increase in electrochemical activity. When x = 0.5 (Na_1.5_Li_0.5_FePO_4_F/C), the oxidation and reduction peaks are the sharpest, and the potential difference between the two pairs of redox peaks is small, indicating that the polarization is small. There are two sets of corresponding redox peaks in the CV curves of the four materials, indicating that the electrodes have good reversibility. According to the Na_2_FePO_4_F electrode reaction mechanism, these two pairs of redox peaks may represent two-phase transition processes, namely Na_2_Fe(II)PO_4_F ↔ Na_1.5_FePO_4_F and Na_1.5_FePO_4_F ↔ NaFe(III)PO_4_F. Figure S1e,f show GCD curves and the first-cycle charge–discharge curves of Na_1.5_Li_0.5_FePO_4_F/C. It can be clearly seen that there is a clear charge–discharge platform at different current densities, which is consistent with the CV curve results.

**Figure 5:**
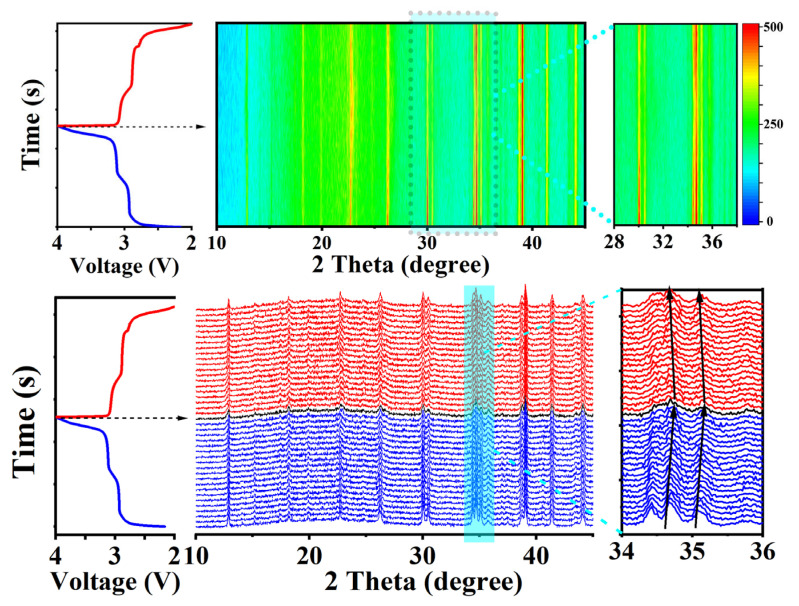
In situ XRD patterns of the Na_1.5_Li_0.5_FePO_4_F/C electrode under different charge/discharge states (blue lines is charge states, black arrows is charging is complete, red lines is discharge states).

Figure 4d shows an AC impedance diagram of the Na_2−x_Li_x_FePO_4_F/C (0 ≤ x ≤ 2) cathode materials. A semicircle in the high- to mid-frequency region and a sloping line in the low-frequency region make up the Nyquist plot. The radius of the semicircle represents the charge transfer resistance (R_ct_) and is related to the charge transfer between the electrolyte and the active material. The intercept of the Z axis corresponds to the Ohmic resistance (R_S_), which includes the contact resistance of the material and the resistance of the electrolyte. The low-frequency slashes represent the Warburg impedance (Zw) and are related to Na^+^ diffusion in the active material [17,18]. Na_1.5_Li_0.5_FePO_4_F has the lowest charge transfer resistance of 172 Ω. The crystal-structure evolution during the sodium-ion insertion/extraction processes was investigated using the in situ XRD technique (Figure 5). The Na-rich Na_1.5−α_Li_0.5_FePO_4_F/C phase and Na-poor Na_β_Li_0.5_FePO_4_F/C phase are clearly observed in the in situ XRD patterns. Obviously, the Na_1.5−α_Li_0.5_FePO_4_F/C peaks disappear upon desodiation and are restored after the sodiation process. On the contrary, Na_β_Li_0.5_FePO_4_F/C peaks form in the initial stage and grow and disappear during the desodiation and sodiation processes, respectively. Moreover, the (133) peak (2*θ =* 34.5) shifts to higher angles during the desodiation process, indicating that the d-spacings decrease during the extraction of the sodium ions. After fully charging, all of the peaks return to their original positions, manifesting excellent structure reversibility. Therefore, the intrinsic sodium storage mechanism and the reason for the excellent cycling stability of Na_1.5_Li_0.5_FePO_4_F/C were revealed using the operando XRD technique [19,20].

The faster the sodium ion diffusion rate, the better the electrochemical performance. Figure S2 shows the electrochemical kinetics of the ten composites after fitting, and Table S3 shows the sodium ion diffusion coefficients. The best sodium ion diffusion coefficient is found with Na_1.5_Li_0.5_FePO_4_F/C (1.38 × 10^−14^ cm^2^·s^−1^), and the slowest diffusion coefficient is found with Li_2_FePO_4_F/C (5.39 × 10^−17^ cm^2^·s^−1^). The sizes of the diffusion coefficients are consistent with the rate performance results shown in Table S4. The results show that the appropriate sodium and lithium contents can activate the carbon layer, and the optimal sodium and lithium contents can achieve the optimal sodium ion diffusion coefficient.

**Figure 6:**
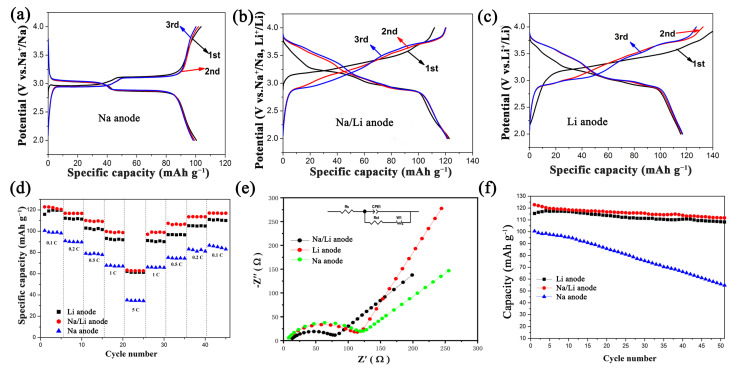
Charge–discharge voltage profiles of batteries with (**a**) Na anode, (**b**) Li anode, and (**c**) Na/Li anode, as well as (**d**) rate capability, (**e**) Nyquist plots, and (**f**) cycling performance of three batteries.

Figure 6a–c show the electrochemical performance of Na_1.5_Li_0.5_FePO_4_F/C as a positive electrode in lithium/sodium-ion batteries. By comparing the charge–discharge voltage profiles of the three batteries, it can be concluded that the Na_1.5_Li_0.5_FePO_4_F/C sample has a better specific capacity as a lithium/sodium hybrid ion battery. Figure 6d shows the electrochemical rate capability of a Na_1.5_Li_0.5_FePO_4_F/C cathode at different current densities. At a current density of 0.1 C, the reversible capacity of Na_1.5_Li_0.5_FePO_4_F/C reached 122.7 mAh g^−1^. Even at a high current density of 5.0 C, there was a significant discharge capacity of 63.3 mAh g^−1^, showing excellent amplification performance. After the current density was reduced to 0.1 C, the capacity increased again to 120.3 mAh g^−1^, which was very close to the capacity at the same current density in the previous stage, indicating that the Na_1.5_Li_0.5_FePO_4_F/C material has an excellent reversible capacity. Figure 6e shows a Nyquist diagram of a Na_1.5_Li_0.5_FePO_4_F/C cathode lithium/sodium hybrid ion battery in the frequency range of 0.01~100 kHz. The high-frequency region is semicircular, and the low-frequency region is diagonal. The results show that the series resistance (Rs) and charge transfer resistance (Rct) of lithium/sodium-ion batteries are the smallest. Compared with other batteries, lithium/sodium hybrid batteries have higher electronic conductivity. More importantly, Na_1.5_Li_0.5_FePO_4_F/C also exhibited excellent cyclic stability when tested at a current density of 0.1 C (Figure 6f). It can be seen that the reversible capacity of the Na_1.5_Li_0.5_FePO_4_F/C material after 50 cycles was as high as 111.8 mAh g^−1^, which was equivalent to 92% of the discharge capacity of the second cycle. This further proved that the material has excellent cycle stability in lithium–sodium hybrid batteries.

## 3. Conclusions

In summary, a high-temperature solid-phase method was used to create a series of carbon-coated cathode materials with the general formula Na_2−x_Li_x_FePO_4_F/C (0 ≤ x ≤ 2), and XRD and TEM were used to characterize the morphology of the as-prepared materials. The results show that the Na_1.5_Li_0.5_FePO_4_F/C material synthesized at a reaction temperature of 600 °C with a calcination time of 10 h had the best electrochemical performance at a rate of 0.1 C. Tests showed that the electrochemical performance of the material gradually improved with the continuous addition of lithium, and when x = 0.5, the performance was optimal, with a discharge-specific capacity in the first week of 122.9 mAh·g^−1^. The discharge-specific capacity remained at 118.2 mAh·g^−1^ after 100 cycles, the Coulombic efficiency was close to 100%, and the sodium ion diffusion coefficient was 1.38 × 10^−14^. The specific capacity of Na_1.5_Li_0.5_FePO_4_F/C can reach 122.7 mAh g^−1^ at 0.1 C under the design conditions of a lithium–sodium blender, and the capacity retention is high (92% after 50 cycles). The design and implementation of high-performance electrochemical energy storage of LNIBs is of great significance and will become a research hotspot of secondary hybrid ion batteries.

## Data Availability

Data are contained within the article and supplementary materials.

## Supplementary Materials

The following supporting information can be downloaded at https://www.mdpi.com/article/10.3390/mi15010015/s1, Figure S1. (a–d) CV curves of Na_2−x_Li_x_FePO_4_F/C (x = 0, 0.5, 1, 1.5). (e) GCD curves of the Na_1.5_Li_0.5_FePO_4_F/C at various current densities. (f) The first cycle charge-discharge curves of the Na_1.5_Li_0.5_FePO_4_F/C materials. Figure S2. Relationship between Z’ and ω^−1/2^ in the low-frequency region of all Na_2−x_Li_x_FePO_4_F/C (0 ≤ x ≤ 2) samples. Table S1. Three different combinations of anode and electrolyte. Table S2. Lattice parameters of the Na_2−X_Li_x_FePO_4_F/C (0 ≤ x ≤ 2) samples obtained by solid-state route and chemical and electrochemical Na^+^/Li^+^ exchange. Table S3. Comparison of the electrochemical properties of Na_2−x_Li_x_FePO_4_F/C (0 ≤ x ≤ 2) materials. Table S4. Sodium ion diffusion coefficient D_Na*+*_ for the Na_2−x_Li_x_FePO_4_F/C (0 ≤ x ≤ 2) materials.
