# Supplementary material for: Preparation and Electrochemical Performance of Na2−xLixFePO4F/C Composite Cathode Materials with Different Lithium/Sodium Ratios
Source: Micromachines (Basel). 2023 Dec 21;15(1):15. doi: 10.3390/mi15010015 (PMC10821228; doi:10.3390/mi15010015)
Supplement: Supplementary file 1 [file micromachines-15-00015-s001.zip › micromachines-2709449-supplementary.pdf]

Supporting Information

Preparation and Electrochemical Performance of $Na_{2-x}Li_xFePO_4F/C$ Composite Cathode Materials with Different Lithium/Sodium Ratios

Lei Wang [1,2], Hualing Tian [1,2], Xiang Yao [1,2], Yanjun Cai [1,2], Ziwei Gao [1,2,3,4,*] and Zhi Su [1,2,5,*]

[1] College of Chemistry and Chemical Engineering, Xinjiang Normal University, Urumqi 830054, China

[2] Xinjiang Key Laboratory of Energy Storage and Photoelectrocatalytic Materials, Urumqi 830054, China

[3] Shanxi Key Laboratory of Chemical Reaction Engineering, College of Chemistry & Chemical Engineering, Yan'an University, Yan'an 716000, China

[4] Key Laboratory of Applied Surface and Colloid Chemistry, Ministry of Education, Xi'an Key Laboratory of Organometallic Material Chemistry, School of Chemistry & Chemical Engineering, Shanxi Normal University, Xi'an 710119, China

[5] College of Energy and Chemical Engineering, Xinjiang Institute of Technology, Akesu 843100, China

* Correspondences: suzhixj@163.com (Z.S.), zwgao@snnu.edu.cn (Z.G.)

S1. Materials and Methods

Analytical-grade reagents were used throughout this study.

A mixture of 0.8995 g of ferrous oxalate, 0.4199 g of sodium fluoride, 0.7801 g of sodium dihydrogen phosphate and 5% glucose was added to 25 mL of anhydrous ethanol solution and stirred for 6 h, then ball milled to obtain the Na_2FePO_4F precursor material. To obtain a pure-phase Na_2FePO_4F/C cathode material, the precursor was pre-sintered at 400 °C for 4 h in an argon atmosphere, then heated to 600 °C for 10 h before being naturally cooled to room temperature. The amount of lithium fluoride added to the precursor was determined by the number of moles of lithium doped (X = 0, 0.1, 0.3, 0.5, 0.7, 1, 1.3, 1.5, 1.7, and 2), and the composites were labeled NFPF/Li/1-NFPF/Li/10. By using a high-temperature solid-phase method, a series of $Na_{2-x}Li_xFePO_4F/C$ (0≤X≤2) were then synthesized according to the formula:

$$NaFePO_4 + xLiF + (2-x)NaF = Na_{2-x}Li_xFePO_4F$$

S2. Material Structural Characterization

The samples were analyzed using an X-ray diffractometer (XRD, D/Max-2400, Rigaku) with a Cu Kα radiation source (λ = 1.5418 Å). A scanning electron microscope (SEM, Sigma 300) operating at 20 kV was used to make morphological measurements of the as-prepared materials. TEM was used to make a detailed examination of the material's surface morphology (Japan-JEOL-JEM 2100 F). The valence states of the elements in the materials were determined by X-Ray photoelectron spectroscopy (XPS, Thermo Scientific Escalab 250Xi).

S3. Electrochemical performance tests

To prepare the cathode materials, first, the active material and a binder (PVDF) were mixed in a mass ratio of 1:1 with an appropriate amount of nitrogen methyl pyrrolidone. The resulting slurry was ground with a mortar before being uniformly coated on aluminum foil. Ten-millimeter pole pieces were punched out of the foil and then dried in a vacuum drying oven at 110 °C for 12 h to produce the electrode. Following that, a sodium-ion battery was assembled in a vacuum glove box under an argon atmosphere using a sodium sheet as the negative electrode, a sodium ion electrolyte, and a glass fiber separator.

The cathode of $Na_{1.5}Li_{0.5}FePO_4F/C$ is used to assemble the positive electrode of the battery. Table S1 shows the negative electrode and electrolyte in the following three different combinations.

Table S1. Three different combinations of anode and electrolyte.

Battery	Positive	Negative	Electrolyte
A	$Na_{1.5}Li_{0.5}FePO_4F/C$	Sodium plate	Sodium ion electrolyte
B	$Na_{1.5}Li_{0.5}FePO_4F/C$	Lithium plate	Lithium ion electrolyte
C	$Na_{1.5}Li_{0.5}FePO_4F/C$	half of the Sodium plate and half of the Lithium plate	Sodium ion electrolyte and Lithium ion electrolyte

The assembled button battery was left for 12 h at room temperature before testing. A Neware charge-discharge test system was used to conduct constant current charge-discharge cycle tests on the assembled batteries at 25 °C to determine their specific capacity and cycle rate. Cyclic voltammetry (CV) and electrochemical impedance spectroscopy (EIS) measurements were made using a Shanghai Chenhua electrochemical workstation CHI760D. (The scan rate was 0.1 m $V{\cdot}s^{-1}$, the frequency was 10 Hz to 100 Hz, and the voltage was 2.0 V to 4.0 V).

The diffusion coefficient (D) of sodium ions can be calculated from the low-frequency region using the following formulae:

$$D_{Na+} = \frac{R^2T^2}{2S^2n^4F^4C^2\sigma^2} \qquad (1)$$

$$Z' = R_S + R_{ct}\sigma_{\omega}{}^{-1/2} \qquad (2)$$

where R is the gas constant (8.314 J $mol^{-1}{\cdot}K^{-1}$), T is the absolute temperature (298.15 K), S is the area of the positive electrode sheet (1.76 cm^2), n is the number of electrons transferred in the electrode reaction, F is the Faraday constant (96485 C mol^{-1}), C is the concentration of sodium ions (mol cm^{-3}), and δ is the Warburg factor related to Zre and ω [S1].

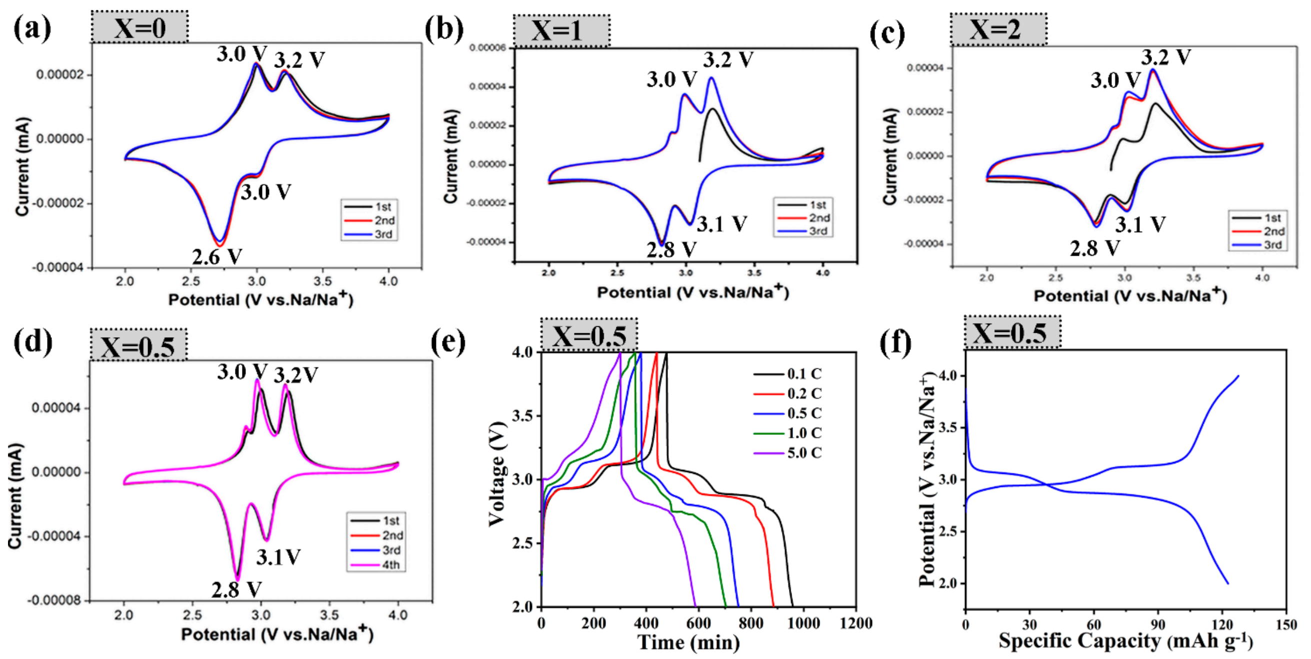

Figure S1. (**a–d**) CV curves of $Na_{2-x}Li_xFePO_4F/C$ (X=0, 0.5, 1, 1.5). (**e**) GCD curves of the $Na_{1.5}Li_{0.5}FePO_4F/C$ at various current densities. (**f**) The first cycle charge-discharge curves of the $Na_{1.5}Li_{0.5}FePO_4F/C$ materials.

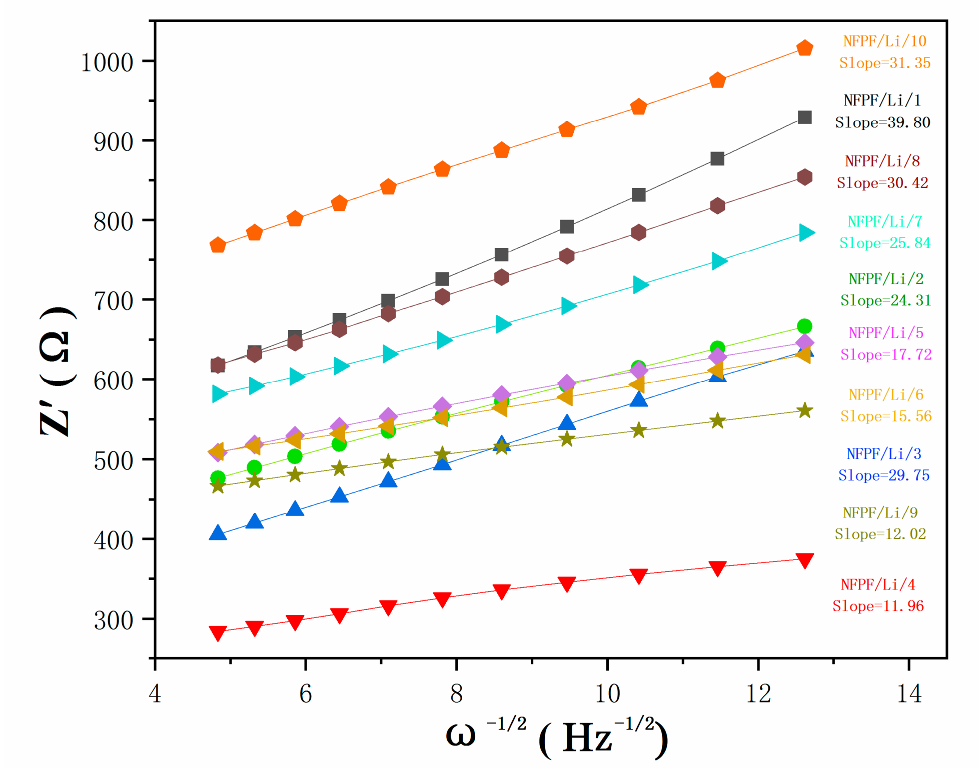

Figure S2. Relationship between Z' and $\omega^{-1/2}$ in the low-frequency region of all $Na_{2-x}Li_xFePO_4F/C$ (0≤X≤2) samples.

Table S2. Lattice parameters of the $Na_{2-x}Li_xFePO_4F/C$ ($0 \le x \le 2$) samples obtained by solid-state route and chemical and electrochemical Na^+/Li^+ exchange.

Sample	Ref.	S. G.	a, Å	b, Å	c, Å	V, Å3
Na_2FePO_4F	this work	Pbcn	5.2190	13.8511	11.7773	851.36
Na_2FePO_4F	[S2]	Pbcn	5.2200	13.8540	11.7792	851.85
$Na_{1.5}Li_{0.5}FePO_4F$	this work	Pnma	10.7992	6.5631	11.5722	820.19
$Na_{1.5}Li_{0.5}FePO_4F$	[S2]	Pnma	10.8052	6.5631	11.4466	811.75
$NaLiFePO_4F$	this work	Pnma	10.9508	6.3987	11.3980	798.67
$NaLiFePO_4F$	[S2]	Pnma	10.9568	6.3959	11.4000	801.7
$Na_{0.3}Li_{1.7}FePO_4F$	this work	Pnma	10.5090	6.4993	11.0485	754.62
$Na_{0.3}Li_{1.7}FePO_4F$	[S2]	Pnma	10.5093	6.4999	11.0483	754.70

Table S3. Comparison of the electrochemical properties of $Na_{2-x}Li_xFePO_4F/C$ ($0 \le X \le 2$) materials.

Different doping ratio	First week discharge specific capacity ($mAh \cdot g^{-1}$)	100-cycle discharge specific capacity ($mAh \cdot g^{-1}$)	Capacity retention rate(%)
Na_2FePO_4F/C	117.7	112.1	95.2%
$Na_{1.9}Li_{0.1}FePO_4F/C$	121.8	98.2	80.6%
$Na_{1.7}Li_{0.3}FePO_4F/C$	122.1	95.6	78.3%
$Na_{1.5}Li_{0.5}FePO_4F/C$	122.9	118.2	96.2%
$Na_{1.3}Li_{0.7}FePO_4F/C$	121.7	90.3	74.2%
$NaLiFePO_4F/C$	121.8	95.0	78.0%
$Na_{0.7}Li_{1.3}FePO_4F/C$	120.0	113.6	94.7%
$Na_{0.5}Li_{1.5}FePO_4F/C$	116.1	104.5	89.9%
$Na_{0.3}Li_{1.7}FePO_4F/C$	111.6	105.1	94.2%
Li_2FePO_4F/C	109.6	96.9	88.4%

Table S4. Sodium ion diffusion coefficient D_{Na+} for the $Na_{2-x}Li_xFePO_4F/C$ (0≤X≤2) materials.

Different doping ratio	D_{Na+} diffusion coefficient($cm^2 \cdot s^{-1}$)
Na_2FePO_4F/C	2.16×10^{-15}
$Na_{1.9}Li_{0.1}FePO_4F/C$	8.22×10^{-16}
$Na_{1.7}Li_{0.3}FePO_4F/C$	3.45×10^{-15}
$Na_{1.5}Li_{0.5}FePO_4F/C$	1.38×10^{-14}
$Na_{1.3}Li_{0.7}FePO_4F/C$	3.45×10^{-15}
$NaLiFePO_4F/C$	2.39×10^{-16}
$Na_{0.7}Li_{1.3}FePO_4F/C$	2.68×10^{-17}
$Na_{0.5}Li_{1.5}FePO_4F/C$	2.82×10^{-14}
$Na_{0.3}Li_{1.7}FePO_4F/C$	4.26×10^{-15}
Li_2FePO_4F/C	5.39×10^{-17}

References

[S1] D. J. Park, R. Rajagopal, K.S. Ryu. Effects of Zr doping to improve ionic conductivity and lithium-diffusion kinetics of β-$LiVO_PO_4$ cathode material, J. Ind. Eng. Chem. 83 (2020) 260-270.

[S2] N.V. Kosova, V.R. Podugolnikov, I.A Bobrikov, A.M. Balagurov, Crystal Structure and Electrochemistry of $Na_{2-x}LixFePO_4F$ (0≤x≤1) New Cathode Materials for Na- and Li-Ion Batteries, ECS Trans. 62 (2014) 67-78.
